# Similar recurrence after curative treatment of HBV-related HCC, regardless of HBV replication activity

**DOI:** 10.1371/journal.pone.0307712

**Published:** 2024-08-26

**Authors:** Mi Na Kim, Beom Kyung Kim, Heejin Cho, Myung Ji Goh, Yun Ho Roh, Su Jong Yu, Dong Hyun Sinn, Soo Young Park, Seung Up Kim

**Affiliations:** 1 Department of Internal Medicine, Yonsei University College of Medicine, Seoul, Republic of Korea; 2 Institute of Gastroenterology, Yonsei University College of Medicine, Seoul, Republic of Korea; 3 Yonsei Liver Center, Severance Hospital, Seoul, Republic of Korea; 4 Department of Internal Medicine and Liver Research Institute, Seoul National University College of Medicine, Seoul, Republic of Korea; 5 Department of Medicine, Samsung Medical Center, Sungkyunkwan University School of Medicine, Seoul, Korea; 6 Biostatistics Collaboration Unit, Department of Biomedical Systems Informatics, Yonsei University College of Medicine, Seoul, Republic of Korea; 7 Department of Internal Medicine, Kyungpook National University Hospital, Daegu, Republic of Korea; Kaohsiung Medical University, TAIWAN

## Abstract

**Background and aims:**

Antiviral therapy (AVT) is required in patients with newly diagnosed hepatitis B virus (HBV)-related hepatocellular carcinoma (HCC), if HBV DNA is detectable. We compared the risk of recurrence according to HBV replication activity at the curative treatment of HBV-related HCC.

**Methods:**

Patients with HBV-related HCC who underwent surgical resection or radiofrequency ablation between 2013 and 2018 were enrolled in this retrospective cohort study. Patients were categorized into two groups according to HBV replication activity at the curative treatment of HBV-related HCC (group 1: patients who met the AVT indication for HBV-related HCC due to detectable HBV DNA but did not meet the AVT indication if without HCC; group 2: patients who met the AVT indication, regardless of HCC).

**Results:**

In the entire cohort (n = 911), HCC recurred in 303 (33.3%) patients during a median follow-up of 4.7 years. After multivariate adjustment, group 2 showed a statistically similar risk of HCC recurrence (adjusted hazard ratio [aHR] = 1.18, *P* = 0.332) compared to that of group 1. In addition, group 2 showed statistically similar risks of early (< 2 years; aHR = 1.31) and late (≥ 2 years; aHR = 0.83) recurrence than that of group 1 (all *P*>0.05). Propensity score matching and inverse probability of treatment weighting analysis also yielded similar risks of HCC recurrence between the two groups (all *P*>0.05, log-rank tests).

**Conclusions:**

The risk of HCC recurrence in patients who received curative treatment for newly diagnosed HBV-related HCC was similar regardless of HBV replication activity, if AVT was properly initiated.

## Introduction

Hepatitis B virus (HBV) infection is a major risk factor of hepatocellular carcinoma (HCC) [1–4]. The prognosis of patients with HCC remains dismal, with a five-year overall survival of approximately 10 to 15%, as most cases of HCC are still diagnosed at an advanced stage, which precludes curative treatment [5–7]. Ultimately, the cornerstone for improving overall survival lies in the early application of curative treatments, such as transplantation, resection, or radiofrequency ablation (RFA) [8–10].

However, even after curative treatment, the long-term prognosis of patients with HCC is poor, primarily because of its high rate of recurrence (>50% at 5 years) [11, 12]. High HBV DNA levels increase the risk of HCC recurrence after curative treatment [13–16]. Subsequently, it has been demonstrated that suppressing HBV replication with antiviral therapy (AVT) using nucleos(t)ide analogs reduces the risk of HCC recurrence and prolongs survival after curative treatment [17–23]. A previous meta-analysis reported that AVT reduces the risk of HCC recurrence by up to 41% in patients with HBV-related HCC who underwent curative treatment [24].

Recent studies have demonstrated that the prognosis of patients with HBV-related HCC who underwent curative treatment was similar, regardless of the timing of AVT initiation (i.e., initiation before and after HCC treatment) [25, 26], suggesting that prompt AVT initiation is beneficial even after HCC diagnosis to a similar degree as those who initiated AVT before HCC development. Of these patients with detectable HBV DNA, some already fulfilled the AVT indication regardless of HCC diagnosis, while others did not fulfill the AVT indication, if without HCC diagnosis, but received AVT due to detectable HBV DNA. However, it remains unclear whether the impact of AVT on outcomes after curative treatment of HBV-related HCC differs between the two groups with different HBV replication activities at the curative treatment of HCC.

Accordingly, in this large-scale multicenter, retrospective cohort study, we compared the HCC recurrence after curative treatment for HBV-related HCC according to HBV replication activity at the curative treatment of HCC.

## Methods

### Study subjects

Patients with newly diagnosed HBV-related HCC who received curative treatment (surgical resection or RFA) as the first-line treatment for HCC and those who started AVT using entecavir (ETV) or tenofovir disoproxil fumarate (TDF) as the first-line AVT agent within three months of curative treatment in five academic teaching institutes in South Korea were considered eligible. From January 1, 2013, to December 31, 2018, a total of 4,219 patients were screened.

The exclusion criteria were as follows: (1) age < 19 years, (2) co-infection with hepatitis C virus, (3) decompensated liver cirrhosis (LC), (4) AVT agents other than ETV or TDF, (5) < 6 months of follow-up, (6) HCC recurrence within 6 months, (7) delayed AVT (≥ 3 months after curative treatment), (8) absence of dynamic imaging, and (9) delayed surveillance interval (≥ 7 months) after curative treatment (**S1 Fig**).

This study received approval from the institutional review boards of all participating institutions (Severance Hospital, Seoul National University Hospital, Kyungpook National University Hospital, and Samsung Medical Center). The study protocol adhered to the ethical guidelines of the 1975 Declaration of Helsinki, and the need for written informed consent was waived due to the retrospective nature of the study. The data were accessed for research purposes from September 11, 2022 to January 11, 2023. Additionally, access to information that could potentially identify individual participants post data collection was secured.

### AVT

AVT indication was in accordance with the chronic hepatitis B (CHB) treatment guideline of the Korean Association for the Study of the Liver and reimbursement guideline of the National Health Insurance Service of South Korea during study period (**S1 Table**) [27, 28]. Briefly, AVT was initiated for CHB patients with (1) HBV DNA ≥20,000 IU/mL for hepatitis B e antigen (HBeAg) positive or HBV DNA ≥2,000 IU/mL for HBeAg negative, and (2) aspartate aminotransferase (AST) or alanine aminotransferase (ALT) ≥ 2x the upper limit of normal (ULN). For patients with compensated LC, AVT was initiated with (1) HBV DNA ≥2,000 IU/mL, and (2) AST or ALT ≥ 1x ULN before September 1 2015 [27], or regardless of AST or ALT after September 1, 2015 [28–30]. For patients with HCC, AVT was initiated with HBV DNA (≥20 IU/mL) regardless of AST or ALT. The ULN of AST and ALT levels was defined as 40 IU/L, according to the reimbursement criteria of South Korea [31].

### Definitions of groups according to HBV replication activity

Patients were categorized into two groups according to HBV replication activity at diagnosis of HCC; **group 1** included patients who met only AVT indication for HBV-related HCC (i.e., HBV DNA ≥20 IU/mL and not indicated for AVT if without HCC) and **group 2** included those who met AVT indication for CHB regardless of HCC (**S2 Fig**). The primary reason for not receiving AVT before HCC development, despite meeting AVT indications, were due to inadequate adherence to regular follow-up for CHB. Most patients in group 2 had insufficient follow-up after being diagnosed with CHB. Additionally, some patients were unaware of their CHB diagnosis until the HCC diagnosis. As a result, these patients lacked regular CHB monitoring and missed opportunities for timely AVT initiation.

### Diagnosis of HCC and LC

HCC diagnosis was based on histological evidence or radiological findings that included dynamic CT and/or MRI showing typical features of HCC (i.e., nodules >1 cm in size with arterial hypervascularity and portal/delayed-phase washout) [11, 12, 32]. LC was clinically defined as follows: (1) ultrasonographic findings suggestive of cirrhosis, including a blunted, nodular liver surface, and/or (2) esophageal or gastric varices [25].

### Study outcomes and follow-up

The primary outcome was HCC recurrence. The same criteria for HCC diagnosis were applied for HCC recurrence. Early HCC recurrence (within 2 years of HCC treatment) and late HCC recurrence (2 years after HCC treatment) were the secondary outcomes [25].

All included patients were followed up with biochemical liver function tests, serum alpha-fetoprotein (AFP) and des-gamma-carboxy-prothrombin (DCP) levels, and imaging with dynamic CT and/or MRI every 3–6 months after curative treatment. No patient underwent adjuvant therapy after surgical resection or RFA during the follow-up period.

### Statistical analysis

The baseline characteristics of the study participants were described as mean ± SD or median (interquartile range [IQR]) for continuous variables and as number (percentage) for categorical variables. The distribution of continuous variables was evaluated using the Kolmogorov-Smirnov test. Continuous variables that were not normally distributed were compared using the Mann-Whitney U test, and Student’s t-test was used to compare normally distributed continuous variables. When less than 20% of cells had an expected frequency <5, the chi-square test was used to compare categorical variables; otherwise, Fisher’s exact test was used.

The cumulative risk of HCC recurrence was computed from the index date to the confirmation of recurrence or the date of the last follow-up using the Kaplan–Meier method. The log-rank test was used to compare each group. We conducted the Schoenfeld residual plots and obtained *P* value from the test to determine if the proportional hazards assumption holds for the groups in the Cox proportional hazards model. Multivariate Cox regression analysis was performed to investigate the independent risks of HCC recurrence according to the groups by calculating adjusted hazard ratios (aHRs) and 95% confidence intervals (CIs). The covariates for multivariable adjustment were age, sex, LC, body mass index, diabetes, hypertension, HBeAg positivity, HBV DNA, AST, ALT, albumin, total bilirubin, prothrombin time, platelet count, AFP, DCP, antiviral agent (ETV vs. TDF), tumor number (single vs. multiple), maximal tumor size, and treatment modality (surgical resection vs. RFA).

Furthermore, propensity score matching (PSM) and inverse probability of treatment weighting (IPTW) were performed to minimize the effect of potential confounders on the comparison of outcomes between groups. Propensity scores were computed using variables such as age, sex, cirrhosis, body mass index, diabetes, hypertension, antiviral agent, HBeAg, AST, ALT, albumin, total bilirubin, prothrombin time, platelet count, AFP, DCP, tumor number, maximum tumor size, and treatment modality. Outcomes were compared between the groups after adjusting for PSM and IPTW. The IPTW method assigns weights to each individual based on the inverse probability of belonging to their observed group. As a result, when applying IPTW, the effective sample size can differ from the actual number of observed events.

Several sensitivity analyses were performed. The main results were validated according to the AVT indication of the American Association for the Study of the Liver (AASLD) [33], European Association for the Study of the Liver (EASL) [34], and Asia-Pacific Association for the Study of the Liver (APASL) [35] (**S1 Table**). In addition, we repeated our analysis in each cohort stratified by treatment modality and presence of LC.

All statistical analyses were performed using SAS, version 9.2 (SAS Institute, Cary, NC), R (V.3.0, http://cran.r-project.org/), and IBM SPSS Statistics for Windows, version 26.0 (IBM, Armonk, NY). Two-tailed *P* values < 0.05 were considered statistically significant.

## Results

### Baseline characteristics

The baseline characteristics of the study population are summarized in **Table 1**. Based on our inclusion and exclusion criteria, 911 patients were finally selected for statistical analysis. Of these, 549 (60.3%) and 362 (39.7%) patients belonged to group 1 and group 2, respectively. Patients in group 1 were significantly older (mean 56.7 vs. 54.5 years) and had a significantly lower proportion of cirrhosis (45.5% vs. 56.6%) HBeAg positivity (24.0% vs. 40.9%), lower HBV DNA (median 2.1 vs. 5.3 log_10_IU/mL), AST (median 32 vs. 39 IU/mL), ALT (median 30 vs. 38 IU/mL), serum albumin (median 4.3 vs. 4.3 g/dL), and AFP levels (median 13.2 vs. 19.0 ng/mL), and a higher proportion of RFA (32.2% vs. 18.5%), than those in the group 2 (all *P*<0.05). The Albumin-Bilirubin (ALBI) grade for patients was calculated [36]. In total, the proportions of patients according to the ALBI grade were as follows: grade 1, 460 patients (50.5%); grade 2, 425 patients (46.7%); and grade 3, 26 patients (2.9%). There was no significant difference between the groups regarding the proportions of the ALBI grades 2 and 3. The proportions of patients according to the Barcelona Clinic Liver Cancer (BCLC) stages [32, 37, 38] were as follows: stage 0, 227 patients (24.9%); stage A, 599 patients (65.8%); and stage B, 85 patients (9.3%). There was no significant difference between the groups regarding the proportion of BCLC stage B. Among patients who underwent surgical resection, those in the group 1 had a significantly lower proportion of microvascular invasion (42.7% vs. 54.6%) and Edmondson-Stein grades 1 and 2 (48.1% vs. 56.9%) (all *P*<0.05).

**Table 1:** Baseline characteristics of the study population.

Variables	Entire population (n = 911)	Group 1 (n = 549, 60.3%)	Group 2 (n = 362, 39.7%)	*P* value^a^
Demographic variables				
Age, years	55.8 ± 9.5	56.7 ± 9.3	54.5 ± 9.6	0.001
Male gender	680 (74.6)	406 (74.0)	274 (75.7)	0.555
Cirrhosis	455 (49.9)	250 (45.5)	205 (56.6)	0.001
Body mass index, kg/m^2^	24.0 [22.3–26.4]	24.0 [22.3–26.4]	24.0 [22.3–26.5]	0.696
Diabetes	128 (14.1)	87 (15.8)	41 (11.3)	0.055
Hypertension	208 (22.8)	123 (22.4)	85 (23.5)	0.705
Laboratory variables				
HBeAg positivity	280 (30.7)	132 (24.0)	148 (40.9)	<0.001
HBV DNA, log_10_IU/mL	3.4 [1.8–5.3]	2.1 [1.3–3.1]	5.3 [4.4–6.2]	<0.001
AST, IU/mL	35 [26–49]	32 [24–45]	39 [30–57]	<0.001
ALT, IU/mL	34 [23–51]	30 [21–45]	38 [28–59]	<0.001
Serum albumin, g/dL	4.3 [4.0–4.6]	4.3 [4.0–4.6]	4.3 [4.0–4.5]	0.035
Total bilirubin, mg/dL	0.7 [0.5–0.9]	0.7 [0.5–0.9]	0.7 [0.5–0.9]	0.208
Prothrombin time, INR	1.1 [1.0–1.1]	1.1 [1.0–1.1]	1.1 [1.0–1.1]	0.968
Albumin-Bilirubin grade, 1/2/3	460(50.5)/425(46.7)/26 (2.9)	284 (51.7)/257 (46.8)/8 (1.5)	176 (48.6)/168 (46.4)/18 (5.0)	
Albumin-Bilirubin grades 2 and 3	451 (49.5)	265 (48.3)	186 (51.4)	0.379
Platelet counts, 1,000/mm^3^	159 [122–201]	157 [120–201]	160 [127–202]	0.446
AFP, ng/mL	14.4 [4.9–131.1]	13.2 [4.4–105.3]	19.0 [5.7–212.9]	0.013
AFP ≥ 20 ng/mL	417 (45.8)	238 (43.4)	179 (49.4)	0.071
DCP, mAU/mL^b^	44.5 [22.5–251.2]	41.0 [22.0–202.7]	49.0 [24.0–322.8]	0.131
Entecavir/tenofovir	459 (50.4)/ 452 (49.6)	282 (51.4)/ 267 (48.6)	177 (48.9)/ 185 (51.1)	0.465
RFA/ surgical resection	244 (26.8)/667 (73.2)	177 (32.2)/ 372 (67.8)	67 (18.5)/ 295 (81.5)	<0.001
BCLC stage, 0/A/B	227 (24.9)/ 599 (65.8)/ 85 (9.3)	143 (26.0)/ 356 (64.8)/ 50 (9.1)	84 (23.2)/ 243 (67.1)/ 35 (9.7)	
BCLC stage B	85 (9.3)	50 (9.1)	35 (9.7)	0.816
Tumor variables				
Multiple tumors	158 (17.3)	98 (17.9)	60 (16.6)	0.619
Maximal tumor size, cm	2.5 [1.8–3.9]	3.5 [2.4–5.1]	2.7 [1.9–4.0]	0.153
Maximal tumor size > 3 cm	351 (38.5)	203 (37.0)	148 (40.9)	0.236
Pathologic findings^c^				
Portal vein invasion	43 (6.4)	25 (6.7)	18 (6.1)	0.747
Microvascular invasion	320 (48.0)	159 (42.7)	134 (54.6)	0.002
Edmondson-Stein grade 1–2	347 (52.0)	179 (48.1)	168 (56.9)	0.023

Data are presented as means ± SD, medians (interquartile ranges), or numbers (%).

Group 1, patients who fulfilled AVT indication only with HCC; Group 2, patients who fulfilled AVT indication.

^a^*P* values according to comparison between group 1 and 2.

^b^DCP values were missing in 21 patients.

^c^Data from patients receiving surgical resection.

AVT, antiviral therapy; HCC, hepatocellular carcinoma; AST, aspartate aminotransferase; ALT, alanine aminotransferase; INR, international normalized ratio; AFP, alpha-fetoprotein; DCP, des-gamma-carboxy-prothrombin; RFA, radiofrequency ablation; BCLC, Barcelona Clinic Liver Cancer.

A total of 534 (58.6%), 107 (11.7%), 141 (15.5%), 104 (11.4%), and 2 (2.7%) patients had HBV DNA levels of ≤4.00, 4.01–5.00, 5.01–6.00, 6.01–7.00, and >7 log_10_IU/mL, respectively (**S3 Fig**).

### Risk of HCC recurrence according to HBV replication activity at the curative treatment

Across the entire cohort, HCC recurred in 303 (33.3%) patients during the follow-up at a median 4.7 (IQR 3.2–5.9) years. Patients with HCC recurrence had a significantly higher proportion of cirrhosis (56.4% vs. 46.7%), diabetes (17.5% vs. 12.3%,), hypertension (26.7% vs. 20.9%), HBeAg positivity (36.3% vs. 28.0%), higher HBV DNA level (median 3.7 vs. 3.3 log_10_IU/mL), higher AST (median 37 vs. 34 IU/L), higher ALT (median 36 IU/L vs. 32 IU/L), lower serum albumin level (median 4.3 vs. 4.3 g/dL), higher AFP (median 22.7 ng/mL vs. 12.1 ng/mL), higher proportion of multiple tumors (24.7% vs. 13.7%), and higher maximal tumor size (median 2.9 vs. 2.4 cm), compared to those of patients without recurrence (all *P*<0.05) (**S2 Table**). In the multivariate analysis, cirrhosis (aHR = 1.28, 95% CI 1.01–1.63), diabetes (aHR = 1.37, 95% CI 1.01–1.87), HBeAg positivity (aHR = 1.48, 95% CI 1.16–1.89), higher AFP level (aHR = 1.00, 95% CI 1.00–1.00), multiple tumors (aHR = 1.49, 95% CI 1.15–1.95), and larger tumor size (aHR = 1.52, 95% CI 1.20–1.93) were independently associated with the increased risk of HCC recurrence (all *P*<0.05) (**S3 Table**).

After full adjustment for the key covariates (Model 3), the group 2 did not show a statistically different risk of HCC recurrence, when compared to the group 1 (aHR = 1.18, 95% CI 0.85–1.64; *P* = 0.332) (**Table 2**). We assessed the proportional hazards assumption to ensure the validity of our Cox proportional hazards model. Based on the Schoenfeld residual test, the group 1 satisfied Cox proportional hazard assumption for the risk of HCC recurrence (*P* = 0.069 compared to the group 2).

**Table 2:** The risk of HCC recurrence, early recurrence, and late recurrence according to the groups.

Groups	Outcome, n (%)	Unadjusted			Multivariate-adjusted								
					Model 1*			Model 2**			Model 3***		
		HR	95% CI	*P* value	HR	95% CI	*P* value	HR	95% CI	*P* value	HR	95% CI	*P* value
HCC recurrence (n = 303)													
Group 1 (n = 549)	161 (29.3)	1 (reference)			1 (reference)			1 (reference)			1 (reference)		
Group 2 (n = 362)	142 (39.2)	1.30	1.04–1.63	0.024	1.33	1.06–1.67	0.015	1.15	0.82–1.60	0.429	1.18	0.85–1.64	0.332
Early recurrence (n = 198)													
Group 1 (n = 549)	101 (18.4)	1 (reference)			1 (reference)			1 (reference)			1 (reference)		
Group 2 (n = 362)	97 (26.8)	1.50	1.14–1.98	0.004	1.53	1.16–2.03	0.003	1.27	0.85–1.90	0.245	1.31	0.88–1.95	0.187
Late recurrence (n = 105)													
Group 1 (n = 549)	60 (10.9)	1 (reference)			1 (reference)			1 (reference)			1 (reference)		
Group 2 (n = 362)	45 (12.4)	0.90	0.61–1.32	0.586	0.91	0.61–1.35	0.634	0.82	0.44–1.53	0.532	0.83	0.45–1.55	0.562

*Model 1: adjusted for age and sex.

**Model 2: adjusted for age, sex, cirrhosis, body mass index, diabetes, hypertension, antiviral agent (entecavir vs. tenofovir), HBeAg positivity, HBV DNA, aspartate aminotransferase, alanine aminotransferase, serum albumin, total bilirubin, prothrombin time, platelet count, alpha-fetoprotein, des-gamma-carboxy-prothrombin, and treatment modality (RFA vs. surgical resection).

***Model 3: adjusted for age, sex, cirrhosis, body mass index, diabetes, hypertension, antiviral agent (entecavir vs. tenofovir), HBeAg positivity, HBV DNA, aspartate aminotransferase, alanine aminotransferase, serum albumin, total bilirubin, prothrombin time, platelet count, alpha-fetoprotein, des-gamma-carboxy-prothrombin, treatment modality (surgical resection vs. RFA), tumor number (single vs. multiple), and maximal tumor size (≤ 3 cm vs. > 3 cm).

Group 1, patients who fulfilled AVT indication only with HCC; Group 2, patients who fulfilled AVT indication.

HCC, hepatocellular carcinoma; HR, hazard ratio; CI, confidence interval; RFA, radiofrequency ablation; AVT, antiviral therapy.

### PSM and IPTW adjustment

**Fig 1:**
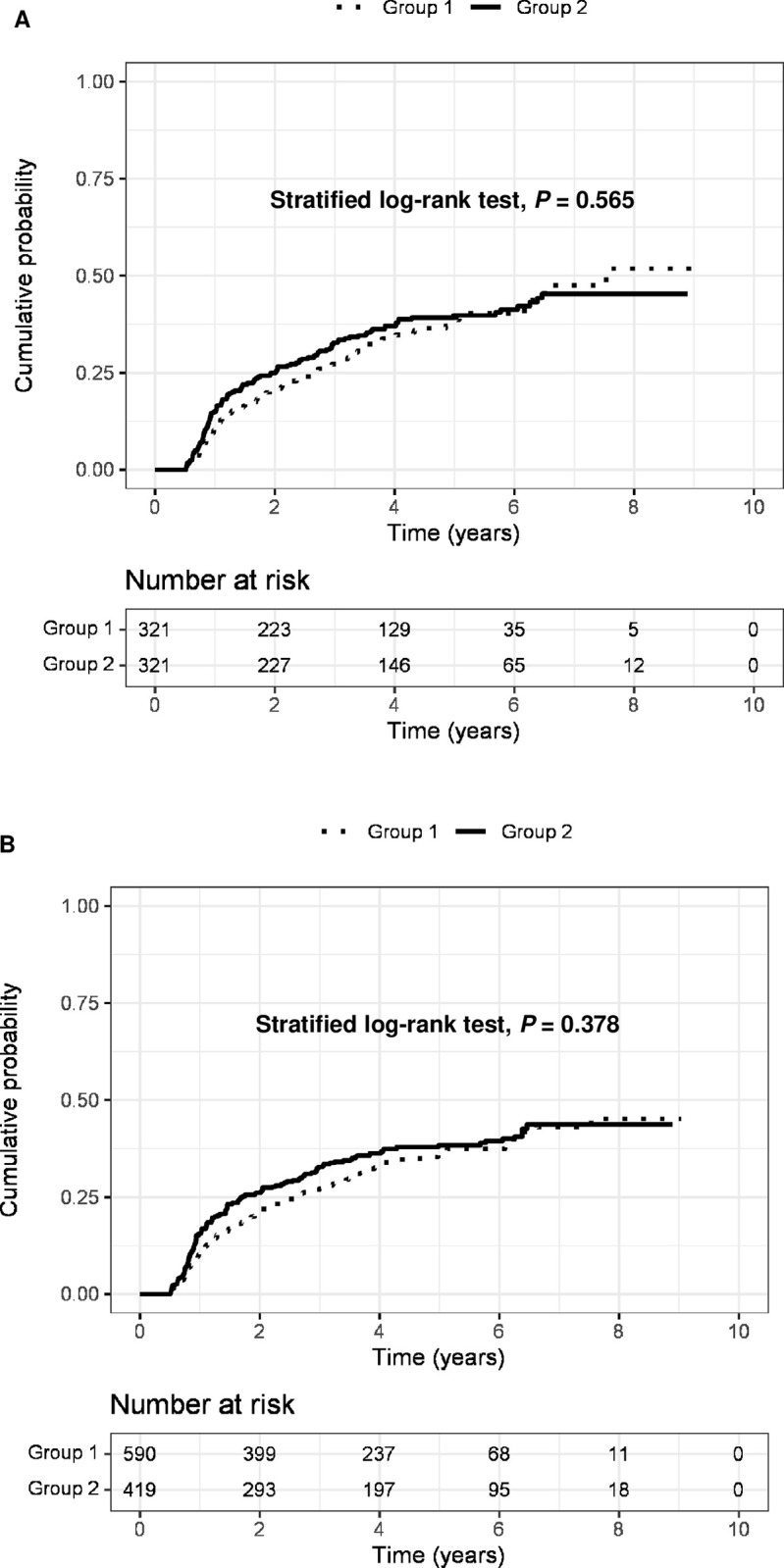
The cumulative probability of HCC recurrence between the group 1 & 2. A, HCC recurrence after propensity score matching adjustment; B, HCC recurrence after inverse probability of treatment weighting adjustment. HCC, hepatocellular carcinoma.

The baseline characteristics of groups 1 and 2 after PSM and IPTW adjustment are shown in **Table 3**. All parameters were well balanced (all *P*>0.05). In PSM analysis with 321 pairs, the cumulative probabilities of HCC recurrence at 1, 3, and 5 years were 11.7%, 27.3%, and 37.9% (incidence rate 11.9 per 100 person-years), respectively in the group 1, and 15.0%, 32.8%, and 39.8% (incidence rate 12.3 per 100 person-years), respectively, in the group 2, without significant difference (*P* = 0.565; **Fig 1A**). Similarly, in the IPTW analysis, similar risk of HCC recurrence was observed between the two groups (*P* = 0.378) (**Fig 1B**).

**Table 3:** Baseline characteristics of groups 1 and 2 after PSM and IPTW adjustment.

Characteristics	PSM				IPTW		
	Group 1 (n = 321)	Group 2 (n = 321)	*P* value	SMD	Group 1 (n = 590)	Group 2 (n = 419)	*P* value
Age, years	54.9 ± 9.0	55.1 ± 9.5	0.777	0.021	55.9 ± 0.4	55.8 ± 0.6	0.929
Male	243 (75.7)	239 (74.5)	0.719	-0.029	75.1 (0.02)	75.3 (0.03)	0.939
Cirrhosis	190 (59.2)	179 (55.8)	0.361	-0.069	51.2 (0.02)	50.9 (0.03)	0.936
Body mass index, kg/m^2^	24.2 ± 3.4	24.3 ± 3.1	0.643	0.037	24.4 ± 0.2	24.3 ± 0.2	0.722
Diabetes	38 (11.8)	37 (11.5)	0.901	-0.010	14.4 (0.02)	14.1 (0.02)	0.909
Hypertension	74 (23.1)	78 (24.3)	0.717	0.030	22.2 (0.02)	23.6 (0.03)	0.651
TDF (vs. ETV)	164 (51.1)	158 (49.2)	0.643	-0.037	49.2 (0.02)	50.0 (0.03)	0.836
HBeAg positivity	108 (33.6)	116 (36.1)	0.458	0.051	30.1 (0.02)	30.5 (0.03)	0.908
AST, IU/mL	44.1 ± 30.3	50.3 ± 50.5	0.017	0.086	45.8 ± 2.8	47.5 ± 2.0	0.632
ALT, IU/mL	43.1 ± 33.6	48.5 ± 38.4	0.012	0.069	46.6 ± 4.2	46.4 ± 2.1	0.958
Albumin, g/dL	4.2 ± 0.5	4.2 ± 0.5	0.974	0.003	4.2 ± 0.03	4.2 ± 0.03	0.885
Total bilirubin, mg/dL	0.8 ± 0.5	0.9 ± 1.1	0.415	0.051	0.8 ± 0.02	0.8 ± 0.04	0.452
Prothrombin time, INR	1.1 ± 0.1	1.1 ± 0.1	0.761	0.023	1.1 ± 0.01	1.1 ± 0.01	0.887
Platelets, 1,000/mm^3^	165.4 ± 65.7	166.1 ± 63.0	0.895	0.011	164.1 ± 2.8	163.9 ± 3.9	0.969
AFP, ng/mL	1528.4 ± 8013.3	1699.6 ± 12573.8	0.838	0.013	1415.7 ± 466.4	1470.4 ± 440.3	0.932
DCP, mAU/mL	1867.2 ± 8336.5	2299.2 ± 9983.1	0.560	0.039	1869.0 ± 392.2	1967.6 ± 421.3	0.864
Multiple tumors	63 (19.6)	55 (17.1)	0.424	-0.066	118.2 (0.02)	118.8 (0.03)	0.846
Maximal tumor size, cm	3.4 ± 2.5	3.5 ± 2.6	0.738	0.024	3.3 ± 0.1	3.3 ± 0.1	0.958
RFA (vs. surgical resection)	68 (21.2)	64 (19.9)	0.666	-0.032	26.4 (0.02)	26.7 (0.03)	0.936

Data of PSM analysis are presented as mean ± SD, medians (interquartile ranges), or numbers (%).

Data of IPTW analysis are presented as means (standard error) or % (standard error).

PSM, propensity score matching; IPTW, inverse probability of treatment weighting; SMD, standardized mean difference; AST, aspartate aminotransferase; ALT, alanine aminotransferase; INR, international normalized ratio; AFP, alpha-fetoprotein; DCP, des-gamma-carboxy-prothrombin; RFA, radiofrequency ablation.

Group 1, patients who fulfilled AVT indication only with HCC; Group 2, patients who fulfilled AVT indication.

When compared to the group 1 as the reference, the HR of the group 2 for HCC recurrence was 1.13 (95% CI 0.87–1.46; *P* = 0.376), indicating no statistical difference between the two groups (**Table 4**).

**Table 4:** The risk of HCC recurrence and mortality according to the groups after PSM and IPTW adjustment.

Groups	Outcome, n (%)	PSM			Outcome, n (%)	IPTW		
		HR	95% CI	*P* value		HR	95% CI	*P* value
HCC recurrence								
Group 1	111 (34.6)	1 (reference)			161 (30.0)	1 (reference)		
Group 2	125 (38.9)	1.072	(0.80–1.45)	0.647	137 (38.7)	1.125	(0.87–1.46)	0.376
Early recurrence								
Group 1	65 (20.2)	1 (reference)			101 (18.8)	1 (reference)		
Group 2	84 (26.2)	1.207	(0.85–1.71)	0.290	93 (26.3)	1.310	(0.95–1.80)	0.095
Late recurrence								
Group 1	46 (14.3)	1 (reference)			60 (11.2)	1 (reference)		
Group 2	41 (12.8)	0.813	(0.48–1.36)	0.432	44 (12.4)	0.758	(0.50–1.15)	0.196

HCC, hepatocellular carcinoma; PSM, propensity score matching; IPTW, inverse probability of treatment weighting; HR, hazard ratio; CI, confidence interval.

Group 1, patients who fulfilled AVT indication only with HCC; Group 2, patients who fulfilled AVT indication.

### Risk of early and late HCC recurrence according to HBV replication activity at the curative treatment

In the entire cohort, 101 patients in the group 1 and 97 patients in the group 2 developed early HCC recurrence. After full adjustment (Model 3), the group 2 did not show a statistically different risk of early HCC recurrence, when compared to the group 1 (aHR = 1.31, 95% CI: 0.88–1.95; *P* = 0.187) (**Table 2**). In addition, 60 patients in the group 1 and 45 in the group 2 developed late HCC recurrence. After full adjustment (Model 3), the group 2 did not show a statistically different cumulative risks of late HCC recurrence compared to the group 1 (aHR = 0.83, 95% CI: 0.45–1.55; *P* = 0.562) (**Table 2**).

**Fig 2:**
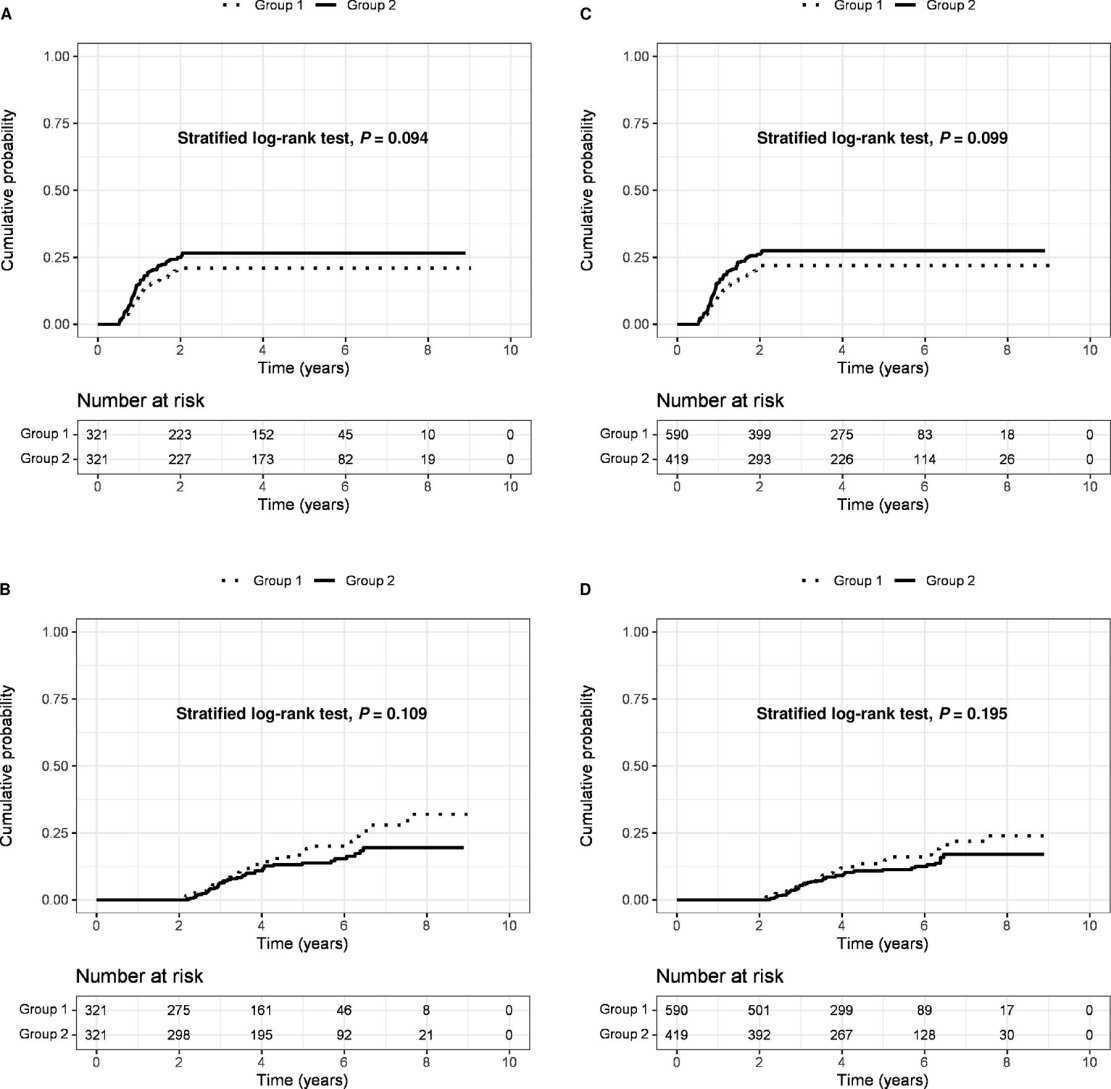
The cumulative probability of early and late HCC recurrence between the group 1 & 2. A, early HCC recurrence after propensity score matching adjustment; B, late HCC recurrence after propensity score matching adjustment; C, early HCC recurrence after inverse probability of treatment weighting adjustment; D, late HCC recurrence after inverse probability of treatment weighting adjustment. HCC, hepatocellular carcinoma.

Similar results were reproduced in PSM and IPTW analyses. The cumulative probabilities of early and late HCC recurrence did not differ between the two groups: (*P* = 0.094) for early recurrence (**Fig 2A**) and (*P* = 0.109) for late recurrence (**Fig 2B**) on PSM, and (*P* = 0.099) for early recurrence (**Fig 2C**) and **(***P* = 0.195) for late recurrence (**Fig 2D**) on IPTW. When compare to the group 1 as the reference, the HRs were 1.31 (95% CI 0.95–1.80; *P* = 0.095) for early recurrence and 0.76 (95% CI 0.50–1.15; *P* = 0.196) for late recurrence, indicating no statistical difference between the two groups (**Table 4**).

### Sensitivity analyses

When the pathological findings were further matched among patients who underwent surgical resection (**S4 Table**), the risk of HCC recurrence, early recurrence, and late recurrence were statistically similar between the two groups (all *P*>0.05) (**S5 Table**).

When patients were categorized into groups 1 and 2 according to the AASLD, EASL, and APASL guidelines (**S1 Table**), the risks of HCC recurrence, early recurrence, and late recurrence were statistically similar between the two groups (all *P*>0.05) (**S6 Table**). In addition, when the patients were divided into two groups with surgical resection and RFA or with cirrhosis and non-cirrhosis, the risk of HCC recurrence, early recurrence, and late recurrence were statistically similar between the two groups (all *P*>0.05) (**S7 and S8 Tables**).

## Discussion

In this multicenter, large-scale cohort study, we found that the risk of HCC recurrence was comparable between the groups categorized by HBV replication activity at the curative treatment of HCC (group 1, patients who met only AVT indication for HBV-related HCC and not indicated for AVT if without HCC, vs. group 2, those who met AVT indication for CHB regardless of HCC) if AVT was properly initiated. Furthermore, the risk of early and late HCC recurrence was also comparable between the groups. These outcomes were reproduced using the Cox regression, PSM, and IPTW analyses.

Persistent HBV replication is a major risk predictor of HCC recurrence after curative surgical resection [14, 39, 40] and RFA [15, 16, 41] in patients with HBV-related HCC who have not received AVT. In a nationwide cohort study from Taiwan involving 4,569 patients with HBV-related HCC who underwent curative liver resections, AVT significantly reduced the risk of HCC recurrence (HR = 0.67), even in patients with a low viral load (HBV DNA level <2,000 IU/mL) [42]. The benefit of AVT in decreasing HCC recurrence has also been observed after RFA [22, 23]. Several meta-analyses have demonstrated that the risk of HCC recurrence and overall mortality after curative treatment is reduced by approximately 40% and 40–70%, respectively [24, 43, 44]. All these studies indicate that effective suppression of HBV replication is crucial for reducing HCC recurrence and improving overall survival in patients with HBV-related HCC, even after curative treatment. However, the efficacy of AVT after curative treatment for HBV-related HCC according to HBV replication activity remains unclear.

Our study has several strengths. First, to the best of our knowledge, our study is the first report on the direct comparison of long-term outcomes of AVT according to HBV replication activity, that is, fulfilment of AVT indication only with HCC at the curative treatment and fulfillment of AVT indication at the curative treatment. We directly compared the efficacy according to the different fulfillment of AVT indication of potent AVT, EVT, and TDF on HCC recurrence after curative treatment.

Second, based on our large cohort size (approximately 900 patients), we were able to separately explore the efficacy of AVT between the two groups for the prevention of early and late recurrence as the secondary outcome. It is generally known that most early recurrence within 2 years of curative treatment results from occult metastasis from the primary tumor [45], whereas late recurrence after 2 years of curative treatment is a de novo second primary tumor spontaneously arising in the remaining liver [46, 47]. The observation that the risk of early recurrence is comparable between the two groups demonstrated that the efficacy of AVT on prevention of HCC recurrence was not influenced by factors associated with tumors and/or treatments. More importantly, since late recurrence is associated with underlying liver conditions, such as cirrhosis and/or active HBV replication [48, 49], our findings imply that AVT can similarly prevent HCC recurrence, even in the high-risk late recurrence group.

Third, our data from real-life cohorts, with a relatively large sample of 911 patients from five independent tertiary academic teaching hospitals, enables the applicability of our findings to routine clinical practice. Furthermore, the number of cases in our cohort, including 303 (33.3%) HCCs during the median follow-up period of 4.7 years ensured statistical reliability. Subgroup analyses using various stratifications demonstrated the robustness of the results. The similarity in outcomes between the two groups was consistent regardless of the recurrence type (early or late), HCC treatment modality (surgical resection or RFA), or the presence of cirrhosis.

Fourth, owing to the potential selection bias in our study, the baseline characteristics that can impact HCC recurrence, including age, sex, cirrhosis, HBV DNA level, were significantly different between groups. Thus, we applied stringent statistical approaches using multivariable adjustment with PSM and IPTW analyses to adjust for these differences. Specifically, in patients who underwent surgical resection, we adjusted for pathological variables that are well-known as risk factors for recurrence, such as portal vein invasion, microvascular invasion, and tumor differentiation [50]. Even after matching, we found the consistent results between patients who underwent surgical resection and those from the entire cohort.

Despite its clinical implications, our study has several limitations. First, although we attempted to overcome the nature of the retrospective design by using several strategies of statistical matching, unmeasured bias and confounding factors could not be avoided. Second, given that this was a multicenter study, variations relative to the techniques of surgery and RFA, and post-treatment care techniques might have occurred across the centers. Finally, our results may not be generalizable to patients of other ethnicities with CHB, as most patients with CHB infection in South Korea have genotype C HBV acquired through vertical transmission.

In conclusion, the risk of HCC recurrence in patients who received curative treatment for newly diagnosed HBV-related HCC was similar regardless of HBV replication activity, if AVT was properly initiated. Thus, although HBV replication activity was not appropriately controlled, initiation of AVT using highly potent agents after curative treatment of HBV-related HCC may be sufficient to reduce the risk of HCC recurrence.

## Supporting information

S1 Fig Study flow diagram. HCC, hepatocellular carcinoma; ETV, entecavir; TDF, tenofovir disoproxil fumarate; AVT, antiviral therapy.
(PDF)

S2 Fig Definitions of group 1 & 2 according to HBV replication activity. HBV, hepatitis B virus.
(PDF)

S3 Fig Proportion of patients in each HBV DNA category at enrollment. (PDF)

S1 Table Indication of antiviral therapy in each guideline. (DOCX)

S2 Table Comparison between patients who developed HCC recurrence and those who did not. (DOCX)

S3 Table Independent predictors of HCC recurrence. (DOCX)

S4 Table Baseline characteristics of groups 1 and 2 after PSM and IPTW adjustment in patients receiving surgical resection. (DOCX)

S5 Table The risk of HCC recurrence and mortality according to the groups in patients receiving surgical resection after PSM and IPTW adjustment. (DOCX)

S6 Table The risk of HCC recurrence, early recurrence, and late recurrence according to the groups (defined by AASLD, EASL, and APASL guidelines). (DOCX)

S7 Table The risk of HCC recurrence, early recurrence, and late recurrence according to the groups in each treatment modality (surgical resection and RFA). (DOCX)

S8 Table The risk of HCC recurrence, early recurrence, and late recurrence according to the groups in each cohort of cirrhosis and non-cirrhosis. (DOCX)

## List of abbreviations

HBV: hepatitis B virus
HCC: hepatocellular carcinoma
RFA: radiofrequency ablation
AVT: antiviral therapy
ETV: entecavir
TDF: tenofovir
CHB: chronic hepatitis B
HBeAg: hepatitis B e antigen
AST: aspartate aminotransferase
ALT: alanine aminotransferase
ULN: upper limit of normal
LC: liver cirrhosis
CT: computed tomography
MRI: magnetic resonance imaging
AFP: alpha-fetoprotein
DCP: des-gamma-carboxy-prothrombin
IQR: interquartile range
aHR: adjusted hazard ratio
CI: confidence interval
PSM: propensity score matching
IPTW: inverse probability of treatment weighting
ALBI: Albumin-Bilirubin
BCLC: Barcelona Clinic Liver Cancer
