# Supplementary figures and images for: Similar recurrence after curative treatment of HBV-related HCC, regardless of HBV replication activity
Source: PLoS One. 2024 Aug 26;19(8):e0307712. doi: 10.1371/journal.pone.0307712 (PMC11346930; doi:10.1371/journal.pone.0307712)

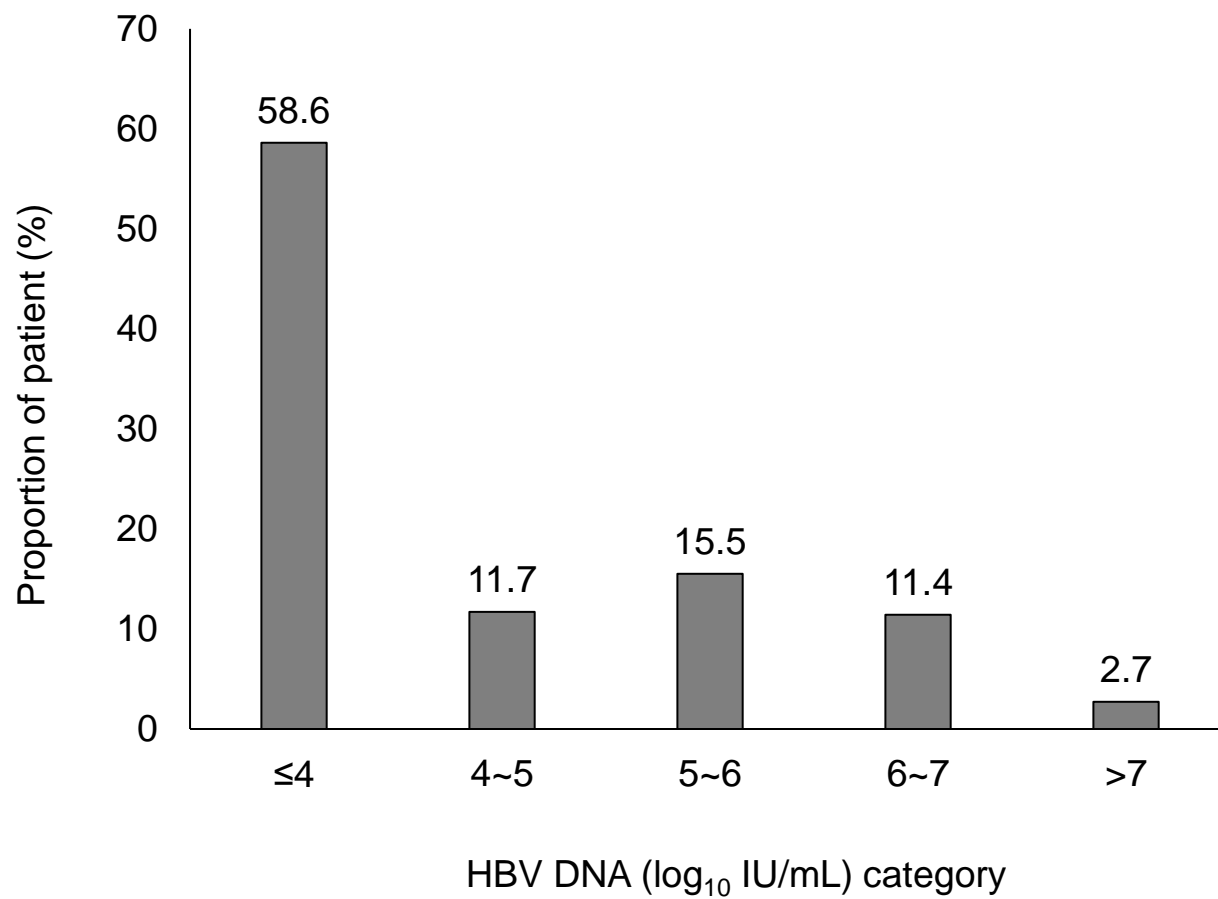

Supplement: S1 Fig — HCC, hepatocellular carcinoma; ETV, entecavir; TDF, tenofovir disoproxil fumarate; AVT, antiviral therapy. (PDF) [file pone.0307712.s001.pdf]
