# Supplementary material for: Similar recurrence after curative treatment of HBV-related HCC, regardless of HBV replication activity
Source: PLoS One. 2024 Aug 26;19(8):e0307712. doi: 10.1371/journal.pone.0307712 (PMC11346930; doi:10.1371/journal.pone.0307712)
Supplement: S2 Fig — HBV, hepatitis B virus. (PDF) [file pone.0307712.s002.pdf]

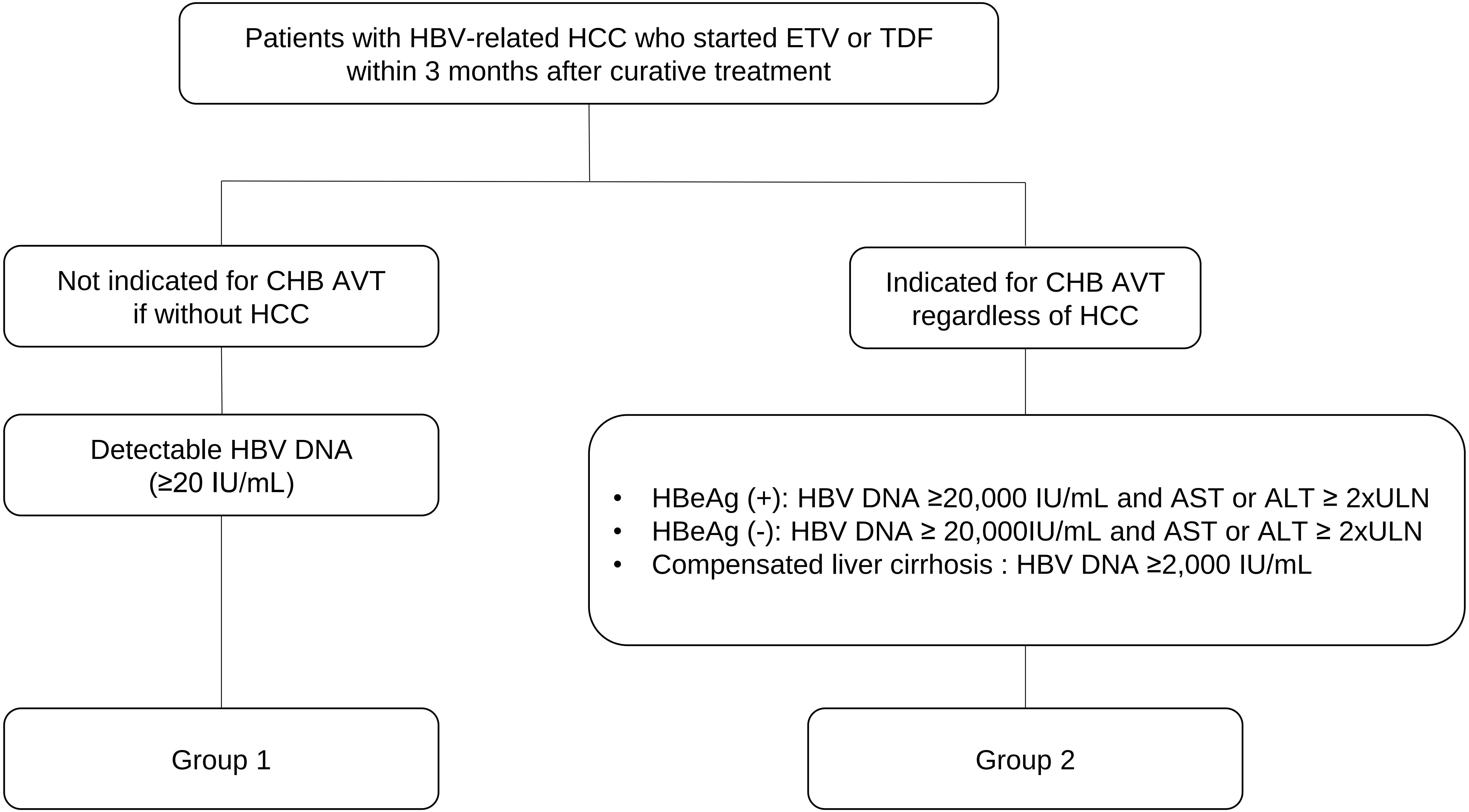
Patients with HBV-related HCC who started ETV or TDF within 3 months after curative treatment
Not indicated for CHB AVT if without HCC
Indicated for CHB AVT regardless of HCC
Detectable HBV DNA (≥20 IU/mL)
• HBeAg (+): HBV DNA ≥20,000 IU/mL and AST or ALT ≥ 2xULN
• HBeAg (-): HBV DNA ≥ 20,000IU/mL and AST or ALT ≥ 2xULN
• Compensated liver cirrhosis : HBV DNA ≥2,000 IU/mL
Group 1
Group 2
