# Supplementary material for: Similar recurrence after curative treatment of HBV-related HCC, regardless of HBV replication activity
Source: PLoS One. 2024 Aug 26;19(8):e0307712. doi: 10.1371/journal.pone.0307712 (PMC11346930; doi:10.1371/journal.pone.0307712)
Supplement: S3 Fig — (PDF) [file pone.0307712.s003.pdf]

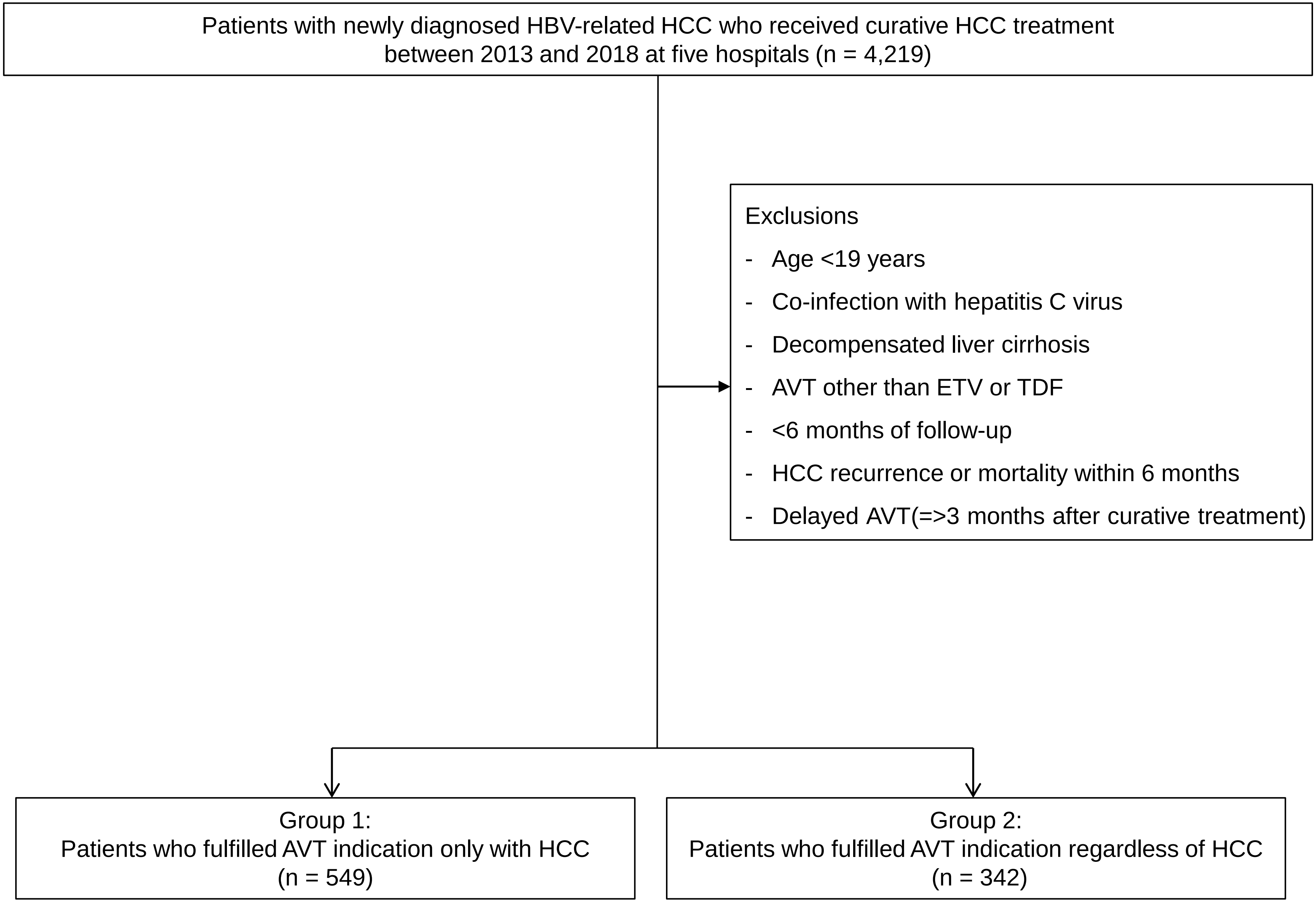
Patients with newly diagnosed HBV-related HCC who received curative HCC treatment between 2013 and 2018 at five hospitals (n = 4,219)
Exclusions
- Age <19 years
- Co-infection with hepatitis C virus
- Decompensated liver cirrhosis
- AVT other than ETV or TDF
- <6 months of follow-up
- HCC recurrence or mortality within 6 months
- Delayed AVT(=>3 months after curative treatment)
Group 1: Patients who fulfilled AVT indication only with HCC (n = 549)
Group 2: Patients who fulfilled AVT indication regardless of HCC (n = 342)
