# Supplementary material for: Similar recurrence after curative treatment of HBV-related HCC, regardless of HBV replication activity
Source: PLoS One. 2024 Aug 26;19(8):e0307712. doi: 10.1371/journal.pone.0307712 (PMC11346930; doi:10.1371/journal.pone.0307712)
Supplement: S1 Table — (DOCX) [file pone.0307712.s004.docx]

| **S1 Table.** Indication of antiviral therapy in each guideline | | | |
| --- | --- | --- | --- |
| Disease category | KASL | AASLD | EASL, APASL |
| Chronic hepatitis |  |  |  |
| HBV DNA, IU/mL |  |  |  |
| HBeAg positive | ≥ 20,000 | ≥ 20,000 | ≥ 2,000 |
| HBeAg negative | ≥ 2,000 | ≥ 2,000 |  |
| ALT, IU/L | > 2 x ULN | > 2 x ULN | > 1 x ULN |
| Compensated cirrhosis |  |  |  |
| HBV DNA, IU/mL | ≥ 2,000 | Detectable | Detectable |
| ALT, IU/L | Any | Any | Any |
| HCC |  |  |  |
| HBV DNA, IU/mL | Detectable | Not available | Not available |
| ALT, IU/L | Any |  |  |
| ULN of ALT | 40 IU/L | 35 U/L for men, 25 U/L for women | 40 IU/L |
| KASL, Korean association for the Study of the Liver; AASLD, American Association for the Study of the Liver; EASL, European Association for the Study of the Liver; APASL, Asia-Pacific Association for the Study of the Liver; HBV, hepatitis B virus; HBeAg, hepatitis B e antigen; ALT, alanine aminotransferase; ULN, upper limit of normal; HCC, hepatocellular carcinoma. | | | |
