# Supplementary material for: Similar recurrence after curative treatment of HBV-related HCC, regardless of HBV replication activity
Source: PLoS One. 2024 Aug 26;19(8):e0307712. doi: 10.1371/journal.pone.0307712 (PMC11346930; doi:10.1371/journal.pone.0307712)
Supplement: S2 Table — (DOCX) [file pone.0307712.s005.docx]

| **S2 Table.** Comparison between patients who developed HCC recurrence and those who did not | | | |
| --- | --- | --- | --- |
| Variables | No recurrence (n=608, 66.7%) | HCC recurrence  (n=303, 33.3%) | *P* value |
| Demographic variables |  |  |  |
| Age, years | 55.6 ± 9.7 | 56.2 ± 9.0 | 0.368 |
| Male gender | 451 (74.2) | 229 (75.6) | 0.647 |
| Cirrhosis | 284 (46.7) | 171 (56.4) | 0.006 |
| Body mass index, kg/m^2^ | 23.9 [22.3-26.3] | 24.1 [22.4-26.6] | 0.484 |
| Diabetes | 75 (12.3) | 53 (17.5) | 0.035 |
| Hypertension | 127 (20.9) | 81 (26.7) | 0.048 |
| Laboratory variables |  |  |  |
| HBeAg positivity | 170 (28.0) | 110 (36.3) | 0.010 |
| HBV DNA, log_10_IU/mL | 3.3 [1.6-5.2] | 3.7 [2.0-5.6] | 0.024 |
| AST, IU/mL | 34 [24-46] | 37 [28-56] | 0.001 |
| ALT, IU/mL | 32 [23-49] | 36 [26-55] | 0.003 |
| Serum albumin, g/dL | 4.3 [4.0-4.6] | 4.3 [4.0-4.5] | 0.035 |
| Total bilirubin, mg/dL | 0.7 [0.5-0.9] | 0.7 [0.5-0.9] | 0.575 |
| Prothrombin time, INR | 1.1 [1.0-1.1] | 1.1 [1.0-1.1] | 0.400 |
| Platelet counts, 1,000/mm^3^ | 160 [125-202] | 155 [119-198] | 0.204 |
| AFP, ng/mL | 12.1 [4.4-97.6] | 22.7 [5.9-242.4] | 0.008 |
| AFP ≥20 ng/mL | 261 (42.9) | 156 (51.5) | 0.015 |
| DCP, mAU/mL^a^ | 52.0 [24.0-347.5] | 52.0 [24.0-347.5] | 0.056 |
| Entecavir/tenofovir | 304 (50.0)/ 304 (50.0) | 155 (51.2)/ 148 (48.8) | 0.743 |
| RFA/surgical resection | 171 (28.1)/ 437 (71.9) | 73 (24.1)/ 230 (75.9) | 0.195 |
| Tumor variables |  |  | <0.001 |
| Multiple tumors | 83 (13.7) | 75 (24.8) |  |
| Maximal tumor size, cm | 2.4 [1.8-3.7] | 2.9 [1.9-4.2] | 0.008 |
| Maximal tumor size > 3 cm | 212 (34.9) | 139 (45.9) | 0.001 |
| Pathologic findings^b^ |  |  |  |
| Portal vein invasion | 12 (6.6) | 6 (5.4) | 0.676 |
| Microvascular invasion | 93 (50.8) | 68 (60.7) | 0.098 |
| Edmondson-Stein grade 1&2 | 107 (58.5) | 61 (54.5) | 0.500 |
| Data are presented as means ± SD, medians (interquartile ranges), or numbers (%). | | | |
| ^a^DCP values were missing in 21 patients. | | | |
| ^b^Data from patients receiving surgical resection. | | | |
| HCC, hepatocellular carcinoma; AVT, antiviral therapy; AST, aspartate aminotransferase; ALT, alanine aminotransferase; INR, international normalized ratio; AFP, alpha-fetoprotein; DCP, des-gamma-carboxy-prothrombin; RFA, radiofrequency ablation. | | | |
