# Supplementary material for: Similar recurrence after curative treatment of HBV-related HCC, regardless of HBV replication activity
Source: PLoS One. 2024 Aug 26;19(8):e0307712. doi: 10.1371/journal.pone.0307712 (PMC11346930; doi:10.1371/journal.pone.0307712)
Supplement: S3 Table — (DOCX) [file pone.0307712.s006.docx]

| **S3 Table**. Independent predictors of HCC recurrence | | | | | |
| --- | --- | --- | --- | --- | --- |
| **Variables** | Univariate analysis | |  | Multivariate analysis | |
|  | HR (95% CI) | *P* value |  | HR (95% CI) | *P* value |
| Group 2 (vs. Group 1) | 1.30 (1.04–1.63) | 0.024 |  | 1.01 (1.00–1.02) | 0.245 |
| Age, years | 1.01 (1.00–1.02) | 0.242 |  |  |  |
| Male gender | 1.08 (0.83–1.40) | 0.58 |  |  |  |
| Cirrhosis | 1.29 (1.03–1.62) | 0.029 |  | 1.28 (1.01–1.63) | 0.04 |
| Body mass index, kg/m^2^ | 1.00 (0.97–1.04) | 0.874 |  |  |  |
| Diabetes | 1.44 (1.07–1.94) | 0.016 |  | 1.37 (1.01–1.87) | 0.046 |
| Hypertension | 1.36 (1.05–1.75) | 0.019 |  | 1.27 (0.97–1.66) | 0.088 |
| HBeAg positivity | 1.35 (1.07–1.71) | 0.012 |  | 1.48 (1.16–1.89) | 0.002 |
| HBV DNA, log_10_IU/mL | 1.06 (1.00–1.12) | 0.065 |  |  |  |
| AST, IU/mL | 1.00 (1.00–1.00) | 0.668 |  |  |  |
| ALT, IU/mL | 1.00 (1.00–1.00) | 0.888 |  |  |  |
| Serum albumin, g/dL | 0.77 (0.61–0.97) | 0.028 |  | 0.88 (0.68–1.13) | 0.32 |
| Total bilirubin, mg/dL | 0.95 (0.80–1.13) | 0.578 |  |  |  |
| Prothrombin time, INR | 0.60 (0.22–1.64) | 0.315 |  |  |  |
| Platelet counts, 1,000/mm^3^ | 1.00 (1.00–1.00) | 0.441 |  |  |  |
| AFP, ng/mL | 1.00 (1.00–1.00) | 0.017 |  | 1.00 (1.00–1.00) | 0.012 |
| DCP, mAU/mL^a^ | 1.00 (1.00–1.00) | 0.056 |  |  |  |
| Entecavir (vs. tenofovir) | 1.06 (0.85–1..33) | 0.621 |  |  |  |
| RFA (vs. surgical resection) | 0.79 (0.60–1.02) | 0.071 |  |  |  |
| Multiple tumors | 1.67 (1.29–2.17) | < 0.001 |  | 1.49 (1.15–1.95) | 0.003 |
| Maximal tumor size > 3 cm | 1.50 (1.20–1.89) | < 0.001 |  | 1.52 (1.20–1.93) | < 0.001 |
| ^a^DCP values were missing in 21 patients. | | | | | |
| Group 1, patients who fulfilled AVT indication only with HCC; Group 2, patients who fulfilled AVT indication. | | | | | |
| HCC, hepatocellular carcinoma; HR, hazard ratio; CI, confidence interval; AST, aspartate aminotransferase; ALT, alanine aminotransferase; INR, international normalized ratio; AFP, alpha-fetoprotein; DCP, des-gamma-carboxy-prothrombin; RFA, radiofrequency ablation; AVT, antiviral therapy. | | | | | |
