# Supplementary material for: Similar recurrence after curative treatment of HBV-related HCC, regardless of HBV replication activity
Source: PLoS One. 2024 Aug 26;19(8):e0307712. doi: 10.1371/journal.pone.0307712 (PMC11346930; doi:10.1371/journal.pone.0307712)
Supplement: S4 Table — (DOCX) [file pone.0307712.s007.docx]

| **S4 Table.** Baseline characteristics of groups 1 and 2 after PSM and IPTW adjustment in patients receiving surgical resection | | | | | | | |
| --- | --- | --- | --- | --- | --- | --- | --- |
| Characteristics | PSM | |  |  | IPTW | |  |
|  | Group 1 (n=244) | Group 2 (n=244) | *P* value | SMD | Group 1 (n=372) | Group 2 (n=295) | *P* value |
| Age, years | 54.3 ± 9.3 | 53.9 ± 9.6 | 0.631 | -0.041 | 54.9 ± 0.5 | 54.9 ± 0.6 | 0.996 |
| Male | 181 (74.2%) | 187 (76.6%) | 0.527 | 0.058 | 74.4 (0.02) | 73.2 (0.03) | 0.753 |
| Body mass index, kg/m^2^ | 24.2 ± 3.3 | 24.1 ± 3.0 | 0.769 | -0.027 | 24.3 ± 0.2 | 24.3 ± 0.2 | 0.910 |
| Diabetes | 32 (13.1%) | 26 (10.7%) | 0.343 | -0.083 | 14.8 (0.02) | 15.1 (0.03) | 0.9438 |
| Hypertension | 69 (28.3%) | 57 (23.4%) | 0.225 | -0.116 | 23.8 (0.02) | 25.5 (0.03) | 0.6556 |
| Tenofovir/Entecavir | 123 (50.4%) | 121 (49.6%) | 0.856 | -0.016 | 49.3 (0.03) | 49.8 (0.03) | 0.910 |
| HBeAg positivity | 69 (28.3%) | 84 (34.4%) | 0.079 | 0.126 | 28.9 (0.03) | 28.9 (0.03) | 0.994 |
| AST, IU/mL | 41.5 ± 29.7 | 43.9 ± 24.6 | 0.283 | 0.038 | 43.8 ± 2.7 | 45.4 ± 2.1 | 0.633 |
| ALT, IU/mL | 41.7 ± 36.3 | 44.8 ± 27.8 | 0.227 | 0.042 | 44.9 ± 3.6 | 46.1 ± 2.4 | 0.767 |
| Albumin, g/dL | 4.3 ± 0.4 | 4.3 ± 0.4 | 0.991 | 0.001 | 4.3 ± 0.03 | 4.3 ± 0.02 | 0.976 |
| Total bilirubin, mg/dL | 0.7 ± 0.3 | 0.7 ± 0.3 | 0.966 | 0.003 | 0.7 ± 0.02 | 0.7 ± 0.02 | 0.882 |
| Prothrombin time, INR | 1.1 ± 0.1 | 1.1 ± 0.1 | 0.965 | 0.004 | 1.1 ± 0.01 | 1.1 ± 0.01 | 0.905 |
| Platelets, 1,000/mm^3^ | 177.1 ± 56.0 | 177.0 ± 61.4 | 0.984 | -0.002 | 176.3 ± 3.3 | 177.4 ± 4.0 | 0.823 |
| AFP, ng/mL | 2037.8 ± 9221.1 | 2068.9 ± 10424.1 | 0.972 | 0.002 | 1979.3 ± 677.3 | 1932.3 ± 633.02 | 0.960 |
| DCP, mAU/mL | 2631.4 ± 9691.2 | 3184.3 ± 12200.7 | 0.583 | 0.046 | 2808.8 ± 642.2 | 2850.3 ± 655.1 | 0.964 |
| Pathologic findings |  |  |  |  |  |  |  |
| Single tumor | 51 (20.9%) | 48 (19.7%) | 0.732 | -0.031 | 122.5 (0.02) | 121.5 (0.03) | 0.797 |
| Maximal tumor size, cm | 4.2 ± 2.8 | 4.2 ± 3.1 | 0.950 | 0.005 | 4.2 ± 0.2 | 4.2 ± 0.2 | 0.992 |
| Portal vein invasion | 12 (4.9%) | 15 (6.1%) | 0.564 | 0.052 | 6.2 (0.01) | 6.7 (0.02) | 0.806 |
| Microvascular invasion | 121 (49.6%) | 125 (51.2%) | 0.695 | 0.033 | 48.0 (0.03) | 47.9 (0.03) | 0.986 |
| Edmondson-Stein grade 1&2 | 135 (55.3%) | 138 (56.6%) | 0.785 | 0.025 | 51.7 (0.03) | 50.8 (0.03) | 0.824 |
| Cirrhosis | 93 (38.1%) | 96 (39.3%) | 0.774 | 0.025 | 38.6 (0.03) | 39.9 (0.03) | 0.753 |
| Data of PSM analysis are presented as means ± SD, medians (interquartile ranges), or numbers (%).  Data of IPTW analysis are presented as means (standard error) or % (standard error). | | | | | | | |
| PSM, propensity score matching; IPTW, inverse probability of treatment weighting; SMD, standardized mean difference; AST, aspartate aminotransferase; ALT, alanine aminotransferase; INR, international normalized ratio; AFP, alpha-fetoprotein; DCP, des-gamma-carboxy-prothrombin. | | | | | | | |
| Group 1, patients who fulfilled AVT indication only with HCC; Group 2, patients who fulfilled AVT indication. | | | | | | | |
