# Supplementary material for: Similar recurrence after curative treatment of HBV-related HCC, regardless of HBV replication activity
Source: PLoS One. 2024 Aug 26;19(8):e0307712. doi: 10.1371/journal.pone.0307712 (PMC11346930; doi:10.1371/journal.pone.0307712)
Supplement: S5 Table — (DOCX) [file pone.0307712.s008.docx]

| **S5 Table.** The risk of HCC recurrence and mortality according to the groups in patients receiving surgical resection after PSM and IPTW adjustment | | | | | | | | | |
| --- | --- | --- | --- | --- | --- | --- | --- | --- | --- |
| Group | Outcome, n (%) | PSM | | |  | Outcome, n (%) | IPTW | | |
|  |  | HR | 95% CI | *P* value |  |  | HR | 95% CI | *P* value |
| HCC recurrence |  |  |  |  |  |  |  |  |  |
| Group 1 (n=549) | 83 (34.3) | 1 (reference) | | |  | 118 (32.7) | 1 (reference) | | |
| Group 2 (n=362) | 91 (37.6) | 1.000 | (0.71–1.42) | >0.999 |  | 108 (37.5) | 0.956 | (0.72–1.28) | 0.761 |
| Early recurrence |  |  |  |  |  |  |  |  |  |
| Group 1 (n=549) | 58 (24.0) | 1 (reference) | | |  | 82 (22.7) | 1 (reference) | | |
| Group 2 (n=362) | 60 (24.8) | 1.000 | (0.68–1.47) | >0.999 |  | 72 (25.0) | 1.003 | (0.71–1.42) | 0.985 |
| Late recurrence |  |  |  |  |  |  |  |  |  |
| Group 1 (n=549) | 25 (10.3) | 1 (reference) | | |  | 36 (10.0) | 1 (reference) | | |
| Group 2 (n=362) | 31 (12.8) | 1.062 | (0.54–2.10) | 0.862 |  | 36 (12.5) | 0.903 | (0.54–1.50) | 0.695 |
| HCC, hepatocellular carcinoma; PSM, propensity score matching; IPTW, inverse probability of treatment weighting; HR, hazard ratio; CI, confidence interval. | | | | | | | | | |
| Group 1, patients who fulfilled AVT indication only with HCC; Group 2, patients who fulfilled AVT indication. | | | | | | | | | |
