# Supplementary material for: Similar recurrence after curative treatment of HBV-related HCC, regardless of HBV replication activity
Source: PLoS One. 2024 Aug 26;19(8):e0307712. doi: 10.1371/journal.pone.0307712 (PMC11346930; doi:10.1371/journal.pone.0307712)
Supplement: S6 Table — (DOCX) [file pone.0307712.s009.docx]

| **S6 Table**. The risk of HCC recurrence, early recurrence, and late recurrence according to the groups (defined by AASLD, EASL, and APASL guidelines) | | | | | | | | | | | | | | | | |
| --- | --- | --- | --- | --- | --- | --- | --- | --- | --- | --- | --- | --- | --- | --- | --- | --- |
| Guidelines  Groups | Outcome, n (%) | Unadjusted | | |  | Multivariate-adjusted | | | | | | | | | | |
|  |  |  |  |  |  | Model 1^*^ | | |  | Model 2^**^ | | |  | Model 3^***^ | | |
|  |  | HR | 95% CI | *P* value |  | HR | 95% CI | *P* value |  | HR | 95% CI | *P* value |  | HR | 95% CI | *P* value |
| AASLD guideline |  |  |  |  |  |  |  |  |  |  |  |  |  |  |  |  |
| HCC recurrence (n=303) |  |  |  |  |  |  |  |  |  |  |  |  |  |  |  |  |
| Group 1 (n=496) | 145 (29.2) | 1 (reference) | | |  | 1 (reference) | | |  | 1 (reference) | | |  | 1 (reference) | | |
| Group 2 (n=415) | 158 (38.1) | 1.28 | 1.02-1.60 | 0.032 |  | 1.28 | 1.02-1.61 | 0.030 |  | 1.04 | 0.68-1.58 | 0.864 |  | 1.06 | 0.69-1.62 | 0.793 |
| Early recurrence (n=198) |  |  |  |  |  |  |  |  |  |  |  |  |  |  |  |  |
| Group 1 (n=496) | 98 (19.8) | 1 (reference) | | |  | 1 (reference) | | |  | 1 (reference) | | |  | 1 (reference) | | |
| Group 2 (n=415) | 100 (24.1) | 1.23 | 0.93-1.63 | 0.143 |  | 1.23 | 0.93-1.63 | 0.139 |  | 0.99 | 0.58-1.67 | 0.965 |  | 1.02 | 0.60-1.73 | 0.953 |
| Late recurrence (n=105) |  |  |  |  |  |  |  |  |  |  |  |  |  |  |  |  |
| Group 1 (n=496) | 47 (9.5) | 1 (reference) | | |  | 1 (reference) | | |  | 1 (reference) | | |  | 1 (reference) | | |
| Group 2 (n=415) | 58 (14.0) | 0.92 | 0.57-1.48 | 0.732 |  | 0.91 | 0.57-1.46 | 0.696 |  | 1.14 | 0.56-2.34 | 0.719 |  | 1.16 | 0.57-2.39 | 0.684 |
| EASL/APASL guideline |  |  |  |  |  |  |  |  |  |  |  |  |  |  |  |  |
| HCC recurrence (n=303) |  |  |  |  |  |  |  |  |  |  |  |  |  |  |  |  |
| Group 1 (n=429) | 125 (29.1) | 1 (reference) | | |  | 1 (reference) | | |  | 1 (reference) | | |  | 1 (reference) | | |
| Group 2 (n=482) | 178 (36.9) | 1.24 | 0.99-1.56 | 0.061 |  | 1.25 | 0.99-1.57 | 0.057 |  | 1.01 | 0.71-1.45 | 0.955 |  | 1.01 | 0.70-1.44 | 0.974 |
| Early recurrence (n=198) |  |  |  |  |  |  |  |  |  |  |  |  |  |  |  |  |
| Group 1 (n=429) | 85 (19.8) | 1 (reference) | | |  | 1 (reference) | | |  | 1 (reference) | | |  | 1 (reference) | | |
| Group 2 (n=482) | 113 (23.4) | 1.20 | 0.90-1.59 | 0.212 |  | 1.20 | 0.91-1.59 | 0.201 |  | 0.94 | 0.60-1.47 | 0.792 |  | 0.95 | 0.61-1.48 | 0.820 |
| Late recurrence (n=105) |  |  |  |  |  |  |  |  |  |  |  |  |  |  |  |  |
| Group 1 (n=429) | 40 (9.3) | 1 (reference) | | |  | 1 (reference) | | |  | 1 (reference) | | |  | 1 (reference) | | |
| Group 2 (n=482) | 65 (13.5) | 1.31 | 0.88-1.94 | 0.182 |  | 1.31 | 0.88-1.94 | 0.186 |  | 1.23 | 0.66-2.30 | 0.510 |  | 1.25 | 0.67-2.32 | 0.486 |
| ^*^Model 1: adjusted for age and sex. | | | | | | | | | | | | | | | | |
| ^**^Model 2: adjusted for age, sex, cirrhosis, body mass index, diabetes, hypertension, HBeAg positivity, HBV DNA, aspartate aminotransferase, alanine aminotransferase, serum albumin, total bilirubin, prothrombin time, platelet count, alpha-fetoprotein, des-gamma-carboxy-prothrombin, antiviral agent (entecavir vs. tenofovir), and treatment modality (RFA vs. surgical resection). | | | | | | | | | | | | | | | | |
| ^***^Model 3: adjusted for age, sex, cirrhosis, body mass index, diabetes, hypertension, HBeAg positivity, HBV DNA, aspartate aminotransferase, alanine aminotransferase, serum albumin, total bilirubin, prothrombin time, platelet count, alpha-fetoprotein, des-gamma-carboxy-prothrombin, antiviral agent (entecavir vs. tenofovir), treatment modality (surgical resection vs. RFA), tumor number (single vs. multiple), and maximal tumor size (≤ 3 cm vs. > 3 cm). | | | | | | | | | | | | | | | | |
| Group 1, patients who fulfilled AVT indication only with HCC; Group 2, patients who fulfilled AVT indication. | | | | | | | | | | | | | | | | |
| HCC, hepatocellular carcinoma; HR, hazard ratio; CI, confidence interval; RFA, radiofrequency ablation; AVT, antiviral therapy. | | | | | | | | | | | | | | | | |
