# Supplementary material for: Similar recurrence after curative treatment of HBV-related HCC, regardless of HBV replication activity
Source: PLoS One. 2024 Aug 26;19(8):e0307712. doi: 10.1371/journal.pone.0307712 (PMC11346930; doi:10.1371/journal.pone.0307712)
Supplement: S7 Table — (DOCX) [file pone.0307712.s010.docx]

| **S7 Table.** The risk of HCC recurrence, early recurrence, and late recurrence according to the groups in each treatment modality (surgical resection and RFA) | | | | | | | | | | | | | | | | |
| --- | --- | --- | --- | --- | --- | --- | --- | --- | --- | --- | --- | --- | --- | --- | --- | --- |
| Groups | Outcome, n (%) | Unadjusted | | |  | Multivariate-adjusted | | | | | | | | | | |
|  |  |  |  |  |  | Model 1^*^ | | |  | Model 2^**^ | | |  | Model 3^***^ | | |
|  |  | HR | 95% CI | *P* value |  | HR | 95% CI | *P* value |  | HR | 95% CI | *P* value |  | HR | 95% CI | *P* value |
| Surgical resection (n=667) |  |  |  |  |  |  |  |  |  |  |  |  |  |  |  |  |
| HCC recurrence (n=230) |  |  |  |  |  |  |  |  |  |  |  |  |  |  |  |  |
| Group 1 (n=372) | 118 (31.7) | 1 (reference) | | |  | 1 (reference) | | |  | 1 (reference) | | |  | 1 (reference) | | |
| Group 2 (n=295) | 112 (38.0) | 1.11 | 0.86-1.44 | 0.416 |  | 1.32 | 0.98-1.78 | 0.072 |  | 1.00 | 0.69-1.46 | 0.994 |  | 1.03 | 0.71-1.49 | 0.883 |
| Early recurrence (n=158) |  |  |  |  |  |  |  |  |  |  |  |  |  |  |  |  |
| Group 1 (n=372) | 82 (22.0) | 1 (reference) | | |  | 1 (reference) | | |  | 1 (reference) | | |  | 1 (reference) | | |
| Group 2 (n=295) | 76 (25.8) | 1.18 | 0.86-1.61 | 0.302 |  | 1.19 | 0.87-1.63 | 0.273 |  | 1.05 | 0.67-1.63 | 0.843 |  | 1.08 | 0.69-1.67 | 0.743 |
| Late recurrence (n=72) |  |  |  |  |  |  |  |  |  |  |  |  |  |  |  |  |
| Group 1 (n=372) | 36 (9.7) | 1 (reference) | | |  | 1 (reference) | | |  | 1 (reference) | | |  | 1 (reference) | | |
| Group 2 (n=295) | 36 (12.2) | 0.98 | 0.62-1.56 | 0.942 |  | 0.98 | 0.62-1.57 | 0.944 |  | 0.99 | 0.47-2.08 | 0.981 |  | 1.01 | 0.49-2.10 | 0.978 |
| RFA (n=244) |  |  |  |  |  |  |  |  |  |  |  |  |  |  |  |  |
| HCC recurrence (n=73) |  |  |  |  |  |  |  |  |  |  |  |  |  |  |  |  |
| Group 1 (n=177) | 43 (24.3) | 1 (reference) | | |  | 1 (reference) | | |  | 1 (reference) | | |  | 1 (reference) | | |
| Group 2 (n=67) | 30 (44.8) | 1.94 | 1.22-3.10 | 0.005 |  | 1.90 | 1.19-3.05 | 0.008 |  | 1.71 | 0.81-3.61 | 0.163 |  | 1.57 | 0.74-3.34 | 0.240 |
| Early recurrence (n=40) |  |  |  |  |  |  |  |  |  |  |  |  |  |  |  |  |
| Group 1 (n=177) | 19 (10.7) | 1 (reference) | | |  | 1 (reference) | | |  | 1 (reference) | | |  | 1 (reference) | | |
| Group 2 (n=67) | 21 (31.3) | 3.16 | 1.70-5.87 | <0.001 |  | 3.04 | 1.62-5.69 | 0.001 |  | 2.49 | 0.93-6.67 | 0.070 |  | 2.29 | 0.85-6.20 | 0.101 |
| Late recurrence (n=33) |  |  |  |  |  |  |  |  |  |  |  |  |  |  |  |  |
| Group 1 (n=177) | 24 (13.6) | 1 (reference) | | |  | 1 (reference) | | |  | 1 (reference) | | |  | 1 (reference) | | |
| Group 2 (n=67) | 9 (13.4) | 0.74 | 0.34-1.59 | 0.435 |  | 0.74 | 0.34-1.60 | 0.439 |  | 0.53 | 0.14-1.98 | 0.345 |  | 0.49 | 0.13-1.84 | 0.289 |
| ^*^Model 1: adjusted for age and sex. | | | | | | | | | | | | | | | | |
| ^**^Model 2: adjusted for age, sex, cirrhosis, body mass index, diabetes, hypertension, HBeAg positivity, HBV DNA, aspartate aminotransferase, alanine aminotransferase, serum albumin, total bilirubin, prothrombin time, platelet count, alpha-fetoprotein, des-gamma-carboxy-prothrombin, and antiviral agent (entecavir vs. tenofovir). | | | | | | | | | | | | | | | | |
| ^***^Model 3: adjusted for age, sex, cirrhosis, body mass index, diabetes, hypertension, HBeAg positivity, HBV DNA, aspartate aminotransferase, alanine aminotransferase, serum albumin, total bilirubin, prothrombin time, platelet count, alpha-fetoprotein, des-gamma-carboxy-prothrombin, antiviral agent (entecavir vs. tenofovir), tumor number (single vs. multiple), and maximal tumor size (≤ 3 cm vs. > 3 cm). | | | | | | | | | | | | | | | | |
| Group 1, patients who fulfilled AVT indication only with HCC; Group 2, patients who fulfilled AVT indication. | | | | | | | | | | | | | | | | |
| HCC, hepatocellular carcinoma; HR, hazard ratio; CI, confidence interval; RFA, radiofrequency ablation; AVT, antiviral therapy. | | | | | | | | | | | | | | | | |
