# Supplementary material for: Similar recurrence after curative treatment of HBV-related HCC, regardless of HBV replication activity
Source: PLoS One. 2024 Aug 26;19(8):e0307712. doi: 10.1371/journal.pone.0307712 (PMC11346930; doi:10.1371/journal.pone.0307712)
Supplement: S8 Table — (DOCX) [file pone.0307712.s011.docx]

| **S8 Table.** The risk of HCC recurrence, early recurrence, and late recurrence according to the groups in each cohort of cirrhosis and non-cirrhosis | | | | | | | | | | | | | | | | |
| --- | --- | --- | --- | --- | --- | --- | --- | --- | --- | --- | --- | --- | --- | --- | --- | --- |
|  |  |  |  |  |  |  |  |  |  |  |  |  |  |  |  |  |
| Groups | Outcome, n (%) | Unadjusted | | |  | Model 1^*^ | | |  | Model 2^**^ | | |  | Model 3^***^ | | |
|  |  | HR | 95% CI | *P* value |  | HR | 95% CI | *P* value |  | HR | 95% CI | *P* value |  | HR | 95% CI | *P* value |
| Cirrhosis (n=455) |  |  |  |  |  |  |  |  |  |  |  |  |  |  |  |  |
| HCC recurrence (n=171) |  |  |  |  |  |  |  |  |  |  |  |  |  |  |  |  |
| Group 1 (n=250) | 85 (34.0) | 1 (reference) | | |  | 1 (reference) | | |  | 1 (reference) | | |  | 1 (reference) | | |
| Group 2 (n=205) | 86 (42.0) | 1.29 | 0.95-1.74 | 0.099 |  | 1.32 | 0.98-1.78 | 0.072 |  | 1.38 | 0.84-2.28 | 0.201 |  | 1.40 | 0.87-2.28 | 0.170 |
| Early recurrence (n=107) |  |  |  |  |  |  |  |  |  |  |  |  |  |  |  |  |
| Group 1 (n=250) | 50 (20.0) | 1 (reference) | | |  | 1 (reference) | | |  | 1 (reference) | | |  | 1 (reference) | | |
| Group 2 (n=205) | 57 (27.8) | 1.44 | 0.98-2.10 | 0.062 |  | 1.47 | 1.01-2.15 | 0.047 |  | 1.34 | 0.73-2.48 | 0.348 |  | 1.38 | 0.76-2.51 | 0.297 |
| Late recurrence (n=64) |  |  |  |  |  |  |  |  |  |  |  |  |  |  |  |  |
| Group 1 (n=250) | 35 (14.0) | 1 (reference) | | |  | 1 (reference) | | |  | 1 (reference) | | |  | 1 (reference) | | |
| Group 2 (n=205) | 29 (14.1) | 0.93 | 0.57-1.51 | 0.756 |  | 0.93 | 0.57-1.53 | 0.776 |  | 1.19 | 0.49-2.89 | 0.706 |  | 1.17 | 0.48-2.85 | 0.723 |
| Non-cirrhosis (n=456) |  |  |  |  |  |  |  |  |  |  |  |  |  |  |  |  |
| HCC recurrence (n=132) |  |  |  |  |  |  |  |  |  |  |  |  |  |  |  |  |
| Group 1 (n=177) | 76 (25.4) | 1 (reference) | | |  | 1 (reference) | | |  | 1 (reference) | | |  | 1 (reference) | | |
| Group 2 (n=67) | 56 (35.7) | 1.30 | 0.92-1.84 | 0.138 |  | 1.30 | 0.91-1.86 | 0.144 |  | 1.06 | 0.65-1.72 | 0.822 |  | 1.03 | 0.63-1.67 | 0.912 |
| Early recurrence (n=91) |  |  |  |  |  |  |  |  |  |  |  |  |  |  |  |  |
| Group 1 (n=177) | 51 (17.1) | 1 (reference) | | |  | 1 (reference) | | |  | 1 (reference) | | |  | 1 (reference) | | |
| Group 2 (n=67) | 40 (25.5) | 1.54 | 1.02-2.33 | 0.041 |  | 1.56 | 1.02-2.38 | 0.039 |  | 1.30 | 0.73-2.30 | 0.375 |  | 1.28 | 0.72-2.27 | 0.402 |
| Late recurrence (n=41) |  |  |  |  |  |  |  |  |  |  |  |  |  |  |  |  |
| Group 1 (n=177) | 25 (8.4) | 1 (reference) | | |  | 1 (reference) | | |  | 1 (reference) | | |  | 1 (reference) | | |
| Group 2 (n=67) | 16 (10.2) | 0.84 | 0.45-1.59 | 0.594 |  | 0.82 | 0.43-1.58 | 0.553 |  | 0.61 | 0.24-1.56 | 0.300 |  | 0.57 | 0.22-1.49 | 0.253 |
| ^*^Model 1: adjusted for age and sex. | | | | | | | | | | | | | | | | |
| ^**^Model 2: adjusted for age, sex, body mass index, diabetes, hypertension, HBeAg positivity, HBV DNA, aspartate aminotransferase, alanine aminotransferase, serum albumin, total bilirubin, prothrombin time, platelet count, alpha-fetoprotein, des-gamma-carboxy-prothrombin, antiviral agent (entecavir vs. tenofovir), and treatment modality (RFA vs. surgical resection). | | | | | | | | | | | | | | | | |
| ^***^Model 3: adjusted for age, sex, cirrhosis, body mass index, diabetes, hypertension, HBeAg positivity, HBV DNA, aspartate aminotransferase, alanine aminotransferase, serum albumin, total bilirubin, prothrombin time, platelet count, alpha-fetoprotein, des-gamma-carboxy-prothrombin, antiviral agent (entecavir vs. tenofovir), tumor number (single vs. multiple), and maximal tumor size (≤ 3 cm vs. > 3 cm). | | | | | | | | | | | | | | | | |
| Group 1, patients who fulfilled AVT indication only with HCC; Group 2, patients who fulfilled AVT indication. | | | | | | | | | | | | | | | | |
| HCC, hepatocellular carcinoma; HR, hazard ratio; CI, confidence interval; RFA, radiofrequency ablation; AVT, antiviral therapy. | | | | | | | | | | | | | | | | |
